# BEST PROGRAMS FOR CAREGIVING: NEW PUBLIC VERSION FOR FAMILY AND FRIEND CAREGIVERS

**DOI:** 10.1093/geroni/igae098.0032

**Published:** 2024-12-31

**Authors:** Rachel Cannon

**Affiliations:** Chair

## Abstract

A new public version of Best Programs for Caregiving (BPC) launched in 2024 for family and friend caregivers to find and compare evidence-based, dementia caregiving programs available to them in their communities. Over 200 organizations were verified to be delivering one or more of the 47 programs in BPC. Data collected from surveys of developers of BPC programs and the organizations delivering BPC programs were used to understand the reach and characteristics of programs being delivered to caregivers, including information on how caregivers can enroll, types of help and support provided, cost and eligibility requirements, how they are delivered, and adaptations made for diverse communities. Survey results suggest there are more BPC programs available, and these programs have wider reach, than expected, with 25 programs offered to caregivers living anywhere in the US via remote delivery. Additionally, nearly one-fourth of programs (23.2%) were adapted for diverse communities. Along with demonstrating the new version of BPC for caregivers, discussion focuses on how caregivers can use BPC to find programs available to them, challenges encountered with verifying delivery organizations, the importance of developing culturally adapted programs, and how BPC can be used by healthcare systems, medical providers, and community organizations to strengthen implementations of comprehensive dementia care required by the Center for Medicare and Medicaid Innovation’s new payment model “Guiding an Improved Dementia Experience” (GUIDE Model).

